# Attachment styles in Tunisian women with schizophrenia

**DOI:** 10.1192/j.eurpsy.2024.1546

**Published:** 2024-08-27

**Authors:** S. Walha, I. Chaari, M. Sehli, A. Abdelhedi, L. Aribi, F. Charfeddine, N. Mseddi, J. Aloulou

**Affiliations:** Psychiatry « B » department, Hedi Chaker University Hospital, Sfax, Tunisia

## Abstract

**Introduction:**

Attachment refers to the emotional bond between a child and their primary caregiver, reflecting the child’s confidence in the caregiver’s capacity to offer security. Evaluating attachment styles in individuals with schizophrenia spectrum disorders holds significance in pinpointing a potential factor affecting therapeutic relationships. This, in turn, indirectly aids in comprehending the emergence of low adherence as a significant barrier to schizophrenia

**Objectives:**

The goal of this study is to assess attachment styles in women with schizophrenia spectrum disorders.

**Methods:**

We conducted a descriptive and analytical cross-sectional study at the Psychiatry “B” department of Hedi Chaker University Hospital in Sfax, Tunisia, during May and June 2023. Our study involved stabilized female patients diagnosed with either schizophrenia or schizoaffective disorder. We utilized the 26-item Revised Psychosis Attachment Measure (PAM_R) questionnaire translated into Arabic and the Positive and Negative Syndrome Scale (PANSS) score to assess schizophrenic symptoms.

**Results:**

We enrolled a total of 41 female patients in our study, with 65.9% diagnosed with schizophrenia and 34.2% with schizoaffective disorder. The average age of the participants was 49.19 years, ranging from 17 to 79 years old. In terms of attachment styles, avoidant attachment was the most prevalent (60.97%), followed by anxious attachment (24.39%), and disorganized attachment (14.63%). Our study revealed significant associations between avoidant attachment and several factors. Patients who began psychiatric follow-up with hospitalization had a significantly higher level of avoidant attachment compared to those starting with outpatient consultation (p < 0.001). The type of therapy also influenced avoidant attachment, with a significant difference (p < 0.001). Insight into their condition also played a significant role (p < 0.001). Moreover, the age at which psychiatric follow-up began showed a statistically significant correlation with avoidant attachment (Spearman’s ρ = 0.000, p < 0.001). Individuals with higher avoidant attachment tended to have a longer duration of untreated psychosis, supported by a statistically significant positive correlation (Spearman’s ρ = 0.082, p < 0.001). There was also a statistically significant relationship between avoidant attachment and the equivalent dose of chlorpromazine, with a positive correlation (Spearman’s ρ = 0.091, p < 0.001), indicating that individuals with higher avoidant attachment may require higher equivalent doses of chlorpromazine. Finally, higher levels of avoidant attachment were associated with a lower presence of positive symptoms in schizophrenia (Spearman’s ρ = -0.026, p < 0.001).

**Conclusions:**

Insecure attachment is a valuable mechanism for understanding the evolution of schizophrenia spectrum phenomenology and may be a useful target for prophylactic interventions.

**Disclosure of Interest:**

None Declared

